# Quantitative analysis of peri-implant collagen fiber orientation around external hexagon and Morse taper implant connections: A dog model study

**DOI:** 10.4317/jced.64278

**Published:** 2026-07-29

**Authors:** Daniel Sartorelli Marques de Castro, Jefferson David Melo de Matos, Jozely Francisca Mello Lima, Maria Angelica Rehder de Araujo, Mário Alexandre Coelho Sinhoreti, Daher Antonio Queiroz, Adriano Piattelli, Carlos dos Reis Pereira de Araújo

**Affiliations:** 1 Professor of Prosthodontics, Department of Dentistry, University Christus, Fortaleza, Ceará, Brazil; 2 PhD, Department of Biomaterials, Dental Materials and Prosthodontics, Sao Paulo State University (Unesp), Institute of Science and Technology, São José dos Campos, São Paulo, Brazil; 3 Professor of Prosthodontics and Occlusion, Department of Dentistry, Universidade Federal do Ceará UFC, Fortaleza, Ceará, Brazil; 4 Private Surgery Clinician, Bauru, University of São Paulo, Bauru, SP, Brazil; 5 Professor, Department of Restorative Dentistry, Dental Materials Division, Piracicaba Dental School (FOP UNICAMP), Piracicaba, São Paulo, Brazil; 6 DDS, MSc, PhD. Associate Professor, Department of Restorative Sciences and Public Health Dentistry, Nova Southeastern University College of Dental Medicine, Fort Lauderdale, Florida, USA; 7 Adjunct Associate Professor, Department of Restorative Dentistry & Prosthodontics, UTHealth School of Dentistry, Houston, Texas, USA; 8 Department of Medical, Oral and Biotechnological Sciences, University of Chieti-Pescara, Italy; 9 Professor of Prosthodontics, Department of Dentistry, Faculdade de Odontologia de Bauru, FOB/USP, São Paulo, São Paulo, Brasil

## Abstract

**Background:**

Peri-implant soft tissue stability depends on the implant-abutment interface and collagen fiber organization. Unlike natural teeth, peri-implant fibers exhibit a less favorable orientation, potentially weakening the biological seal. Implant connection design may influence this collagen arrangement and tissue integrity.

**Materials and Methods:**

Eighteen immediately loaded implants (n = 9 per group) were placed in nine mongrel dogs. Group I received external hexagon implants, while Group II received Morse taper implants. After a 4-month healing period, animals were euthanized and specimens were processed for histological evaluation. Collagen fiber orientation was analyzed using polarized light microscopy and quantified with ImageJ software. Statistical analysis was performed using the Mann-Whitney U test ( = 0.05).

**Results:**

Group I exhibited predominantly parallel and parallel-oblique collagen fibers, with mean orientations of 19.88° (buccal) and 21.92° (lingual). In contrast, Group II showed mainly oblique and oblique-perpendicular fibers, with mean orientations of 70.20° (buccal) and 54.73° (lingual). No significant differences were observed between buccal and lingual aspects within each group (p > 0.05). However, a significant difference was observed between implant connection types, with Group II presenting a significantly higher proportion of oblique and oblique-perpendicular fibers compared with Group I (p = 0.004; r = 0.71).

**Conclusions:**

Morse taper implants were associated with a more perpendicular collagen fiber orientation, which may enhance the biological seal of peri-implant soft tissues.

## Introduction

Soft tissue stability around dental implants is critical for long-term functional and esthetic outcomes. The integrity of peri-implant mucosa is largely determined by the implant-abutment interface, especially factors such as micromovements, bacterial infiltration, and microgap formation. These conditions have been associated with inflammatory responses, marginal bone changes, and compromise of the peri-implant soft tissue seal (1 - 8). The design of the implant-abutment connection significantly influences peri-implant tissue response. Conical (Morse taper) connections have been associated with greater mechanical stability, decreased bacterial infiltration, and improved preservation of crestal bone compared to external hexagon designs, promoting a more stable peri-implant environment and better soft tissue outcomes (9 - 11). The peri-implant mucosa differs structurally from periodontal tissues due to the absence of the periodontal ligament and a reduced vascular supply. Consequently, the organization of collagen fibers becomes a key factor in maintaining tissue integrity. While collagen fibers in natural teeth are predominantly oriented perpendicular to the root surface, forming an effective protective barrier, peri-implant connective tissue typically exhibits a parallel or circumferential arrangement, which may reduce the effectiveness of this seal (12 - 14). Histological studies indicate that implant design and positioning affect collagen fiber orientation. Variables such as connection geometry, platform switching, and subcrestal placement are linked to greater soft tissue thickness and enhanced connective tissue organization (4 , 15). Conical connections may promote a greater prevalence of oblique and perpendicular collagen fibers, potentially enhancing the biological seal. Morse taper systems, due to their minimized microgap and enhanced mechanical stability, may promote the development of a thicker connective tissue collar and a more favorable collagen fiber orientation, thereby improving protection of peri-implant tissues (16). Although previous histological investigations have compared peri-implant soft tissue characteristics around external hexagon and Morse taper implant connections, quantitative assessment of collagen fiber orientation using polarized light microscopy remains limited. Therefore, the aim of this study was to quantitatively evaluate collagen fiber orientation around dental implants with Morse taper and external hexagon connections. The null hypothesis was that implant-abutment connection design does not influence collagen fiber orientation.

## Materials and Methods

**Figure 1:**
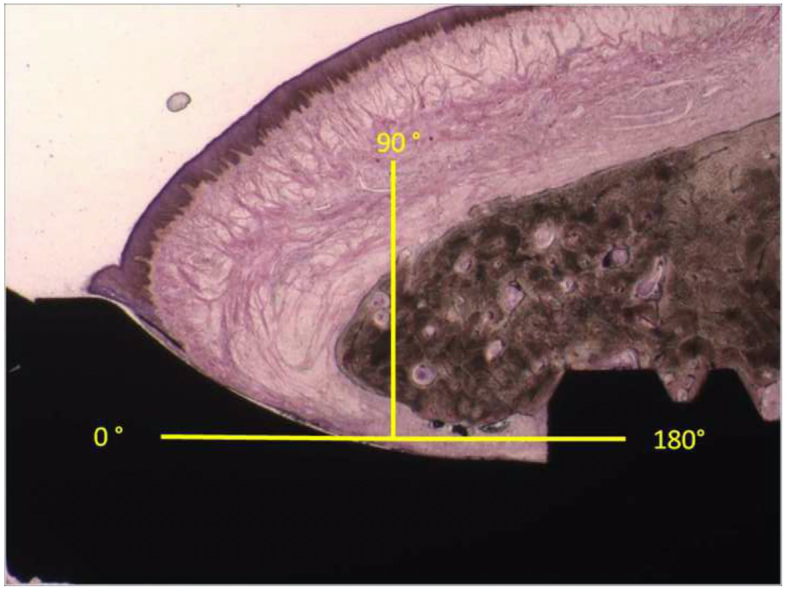
Schematic representation of collagen fiber orientation analysis (0–180°).

1. Study Design and Ethical Considerations This animal study was approved by the Ethics Committee of the School of Dentistry of Bauru, University of São Paulo (São Paulo, Brazil), under protocol number 14/2006. All procedures were conducted in accordance with the European Union Council Directive of November 24, 1986 (86/609/EEC) for the protection of animals used for experimental purposes. 2. Animals and Implant Allocation Nine mongrel dogs (mean weight: 20 kg) were included in this study. A total of 18 dental implants were placed, with each animal receiving two implants, one from each experimental group: Group I: External hexagon implants (3.75 × 11 mm; Osseotite, 3I, Palm Beach Gardens, USA); Group II: Morse taper implants (3.5 × 11 mm; Titamax CM, Neodent, Curitiba, Brazil). 3. Surgical Procedure All animals received intramuscular anesthesia consisting of xylazine (0.1 mg/kg; Anasedan, Vetbrands, Jacareí, SP, Brazil) and ketamine (0.06 mg/kg; Dopalen, Vetbrands, Jacareí, SP, Brazil) prior to the extraction of two maxillary premolars. After a healing period of 4 months, a second surgical procedure was performed. Two implants (one from each group) were placed in each animal in the edentulous premolar region using a full-thickness flap approach and immediately loaded. Implants in Group I were positioned at the crestal level, whereas implants in Group II were placed 2 mm subcrestally. Flaps were sutured using resorbable 3-0 sutures (Techsuture, Bauru, SP, Brazil). Postoperative care included administration of anti-inflammatory medication (flunixin meglumine, 1 mg/kg; Banamine, Intervet Schering-Plough, Cotia, SP, Brazil). A mechanical bite restriction device was used for 7 days to prevent premature loading. During the healing period, animals received periodic sedation with xylazine (0.1 mg/kg) and ketamine (0.06 mg/kg), as well as weekly oral hygiene maintenance. 4. Specimen Collection and Histologic Processing After the healing period, all animals were euthanized, and maxillary segments containing the implants were harvested. Specimens were processed and stained according to previously described protocols. Histological sections were analyzed using a polarized light microscope (Laborlux S, Leica/Leitz, Darmstadt, Germany) coupled with a digital camera (Q Imaging MicroPublisher 3.3 RTV). Images from buccal and lingual aspects were obtained for each specimen to evaluate collagen fiber orientation, origin, and insertion. 5. Histomorphometric analysis Collagen fiber orientation was quantified by calculating the proportion of fibers within specific angular ranges relative to the implant axis. The percentage of each fiber orientation category was determined by dividing the length of fibers within a given angle range by the total length of the analyzed area. Fiber orientation was classified according to Valente et al. (17) as follows: 0°-5°: parallel; 6°-30°: parallel-oblique; 31°-60°: oblique; 61°-85°: oblique-perpendicular; 86°-90°: perpendicular. All measurements were performed using ImageJ software (National Institutes of Health, Bethesda, MD, USA). The analysis was conducted over a 180° area, in which the coronal portion of the implant axis was defined as 0°, and the apical (radicular) direction as 180° (Fig. 1).

1

All analyses were performed by a blinded and calibrated examiner. Examiner calibration was performed before the measurements. Ten randomly selected images were analyzed twice by the same examiner with a two-week interval. Intra-examiner reproducibility was assessed using the intraclass correlation coefficient (ICC). An ICC value above 0.80 was considered indicative of excellent agreement. The intra-examiner ICC was 0.89. 6. Statistical analysis Statistical analysis was performed using the Mann-Whitney U test to compare collagen fiber orientation between groups and between buccal and lingual aspects within each group. The level of statistical significance was set at = 0.05. Effect size (r) was calculated for all Mann-Whitney comparisons. The effect size was calculated as r = Z/N.

## Results

**Figure 2:**
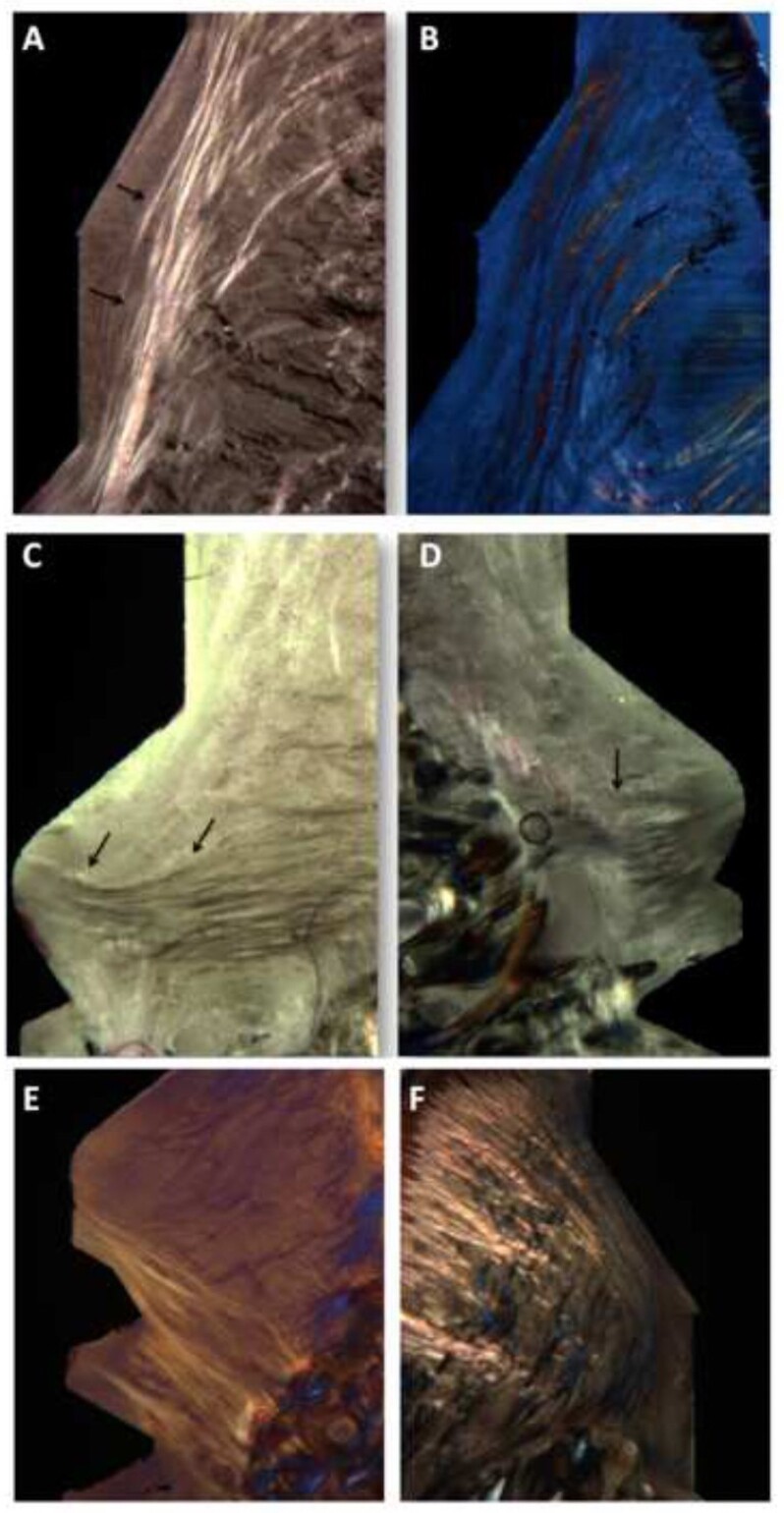
Polarized light microscopy analysis of collagen fibers in Group I (external hexagon). (A, B) Parallel collagen fibers along implant surface. (C, D) Insertion of collagen fibers into implant threads. (E, F) Collagen fibers showing insertion into the implant threads.

All surgical sites healed without complications, and every specimen was deemed suitable for analysis. The comparison between implant connections showed a significant difference (p = 0.004; r = 0.71). 1. Qualitative analysis of collagen fiber direction, origin, and insertion In Group I (external hexagon), collagen fibers originating from the alveolar bone were observed in eight out of nine samples. These fibers were predominantly oriented parallel to the implant surface and its prosthetic component (Fig. 2A,B).

2

**Figure 3:**
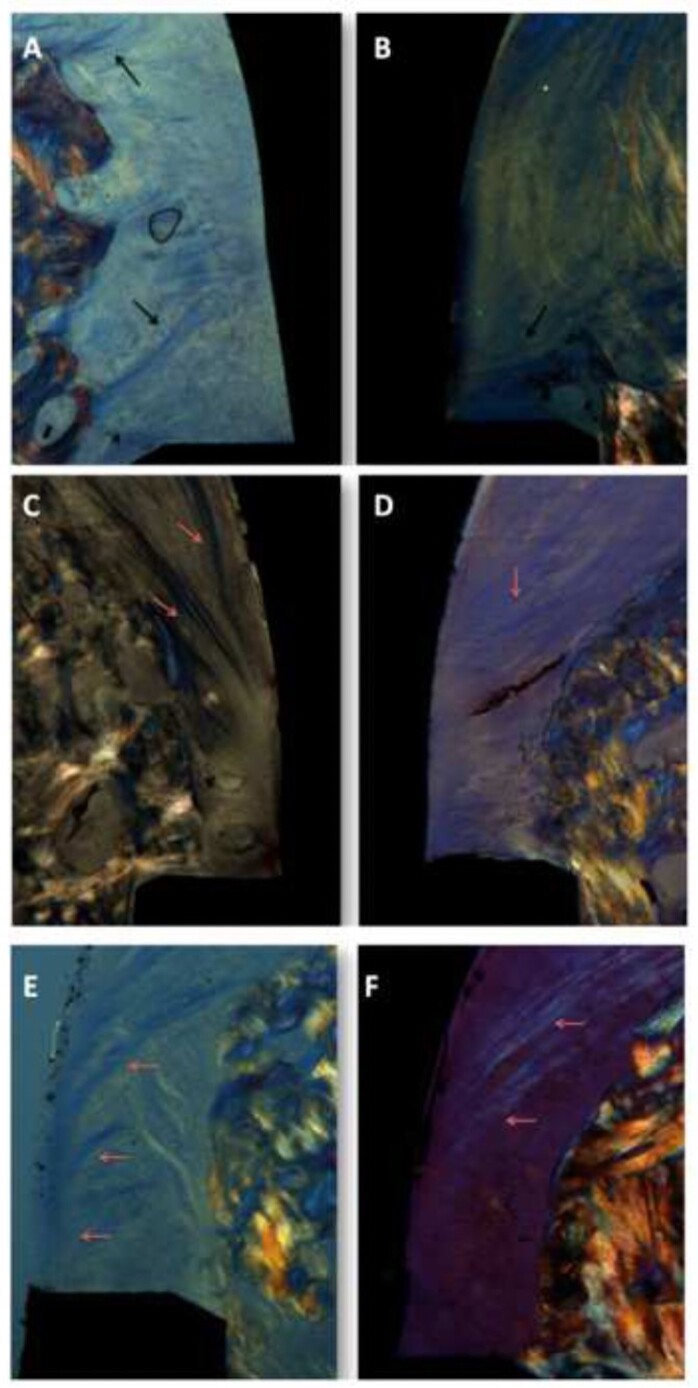
Polarized light microscopy of collagen fibers in Group II (Morse taper). (A–D) Oblique and perpendicular fibers associated with implant and abutment surfaces, showing insertion. (E, F) Fibers originating from the marginal bone crest.

In seven samples, collagen fibers extended from the most external portion of the alveolar crest toward the implant surface, merging with fibers originating from the abutment and oriented toward the implant threads. Perpendicular collagen fibers were identified in only three of the nine samples, located below the prosthetic interface and originating from the marginal crestal bone (Fig. 2C,D). No evidence of direct fiber attachment to the prosthetic component was observed in Group I. However, signs of fiber attachment to implant threads were detected in seven samples (Fig. 2E,F). In Group II (Morse taper), collagen fibers were frequently observed running parallel to the abutment surface and oriented toward the coronal portion of the implant. In six samples, collagen fibers with a perpendicular orientation were identified, distributed laterally along the deeper region of the abutment and near the implant platform. In eight samples, collagen fibers exhibited signs of direct attachment to the abutment surface (Fig. 3A,B).

3

**Figure 4:**
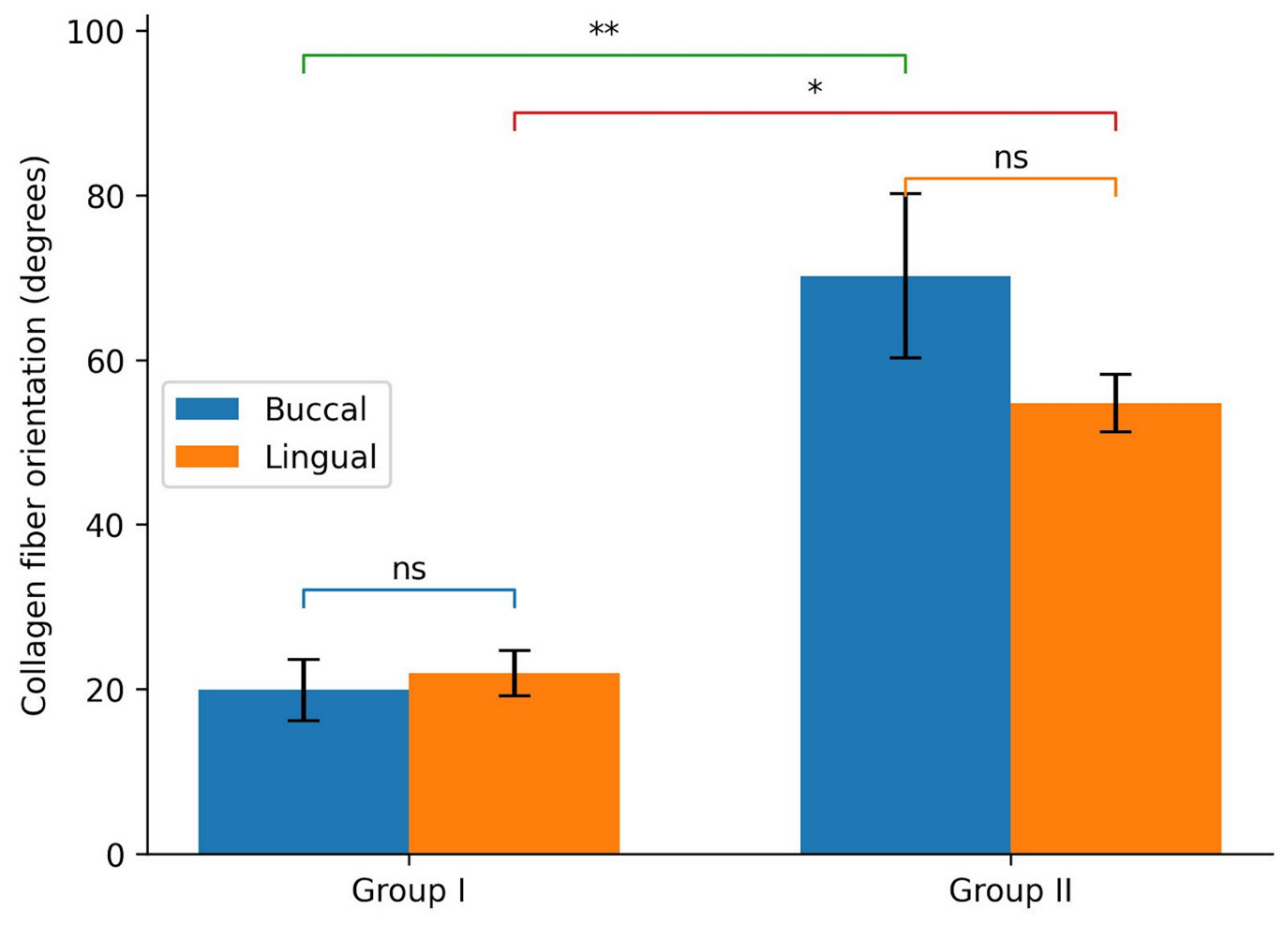
Mean collagen fiber orientation (degrees) in Groups I and II. Error bars: SE. *p < 0.05; **p < 0.01; ns, not significant.

In contrast to Group I, no evidence of fiber attachment to implant threads was observed. Additionally, groups of collagen fibers originating from the marginal bone crest and extending toward the implant were identified, showing predominantly oblique and perpendicular orientations (Fig. 3C,F). A statistically significant difference in collagen fiber orientation was observed between the two implant connection types (Table 1, Fig. 4).

Table 1

4

Comparison between buccal and lingual aspects revealed no statistically significant differences within Group I (p = 0.624) or Group II (p = 0.271). In contrast, comparison between implant connection types demonstrated a statistically significant difference in collagen fiber orientation (p = 0.004; r = 0.71). 2. Quantitative evaluation of collagen fiber orientation In Group I, collagen fibers presented mean angular values ranging from 7.40° to 39.81°. Most fibers were classified as parallel-oblique (88.9%), followed by oblique fibers (11.1%). In Group II, collagen fibers showed mean angular values ranging from 35.54° to 125.70°. Most fibers were classified as oblique (61.1%) and oblique-perpendicular (27.8%). No statistically significant differences were observed between buccal and lingual aspects within either experimental group (p > 0.05). However, significant differences were observed between implant connection types. Group II demonstrated a significantly higher proportion of oblique and oblique-perpendicular collagen fibers compared with Group I (p = 0.004; r = 0.71) (Table 1, Fig. 4).

## Discussion

The present study demonstrated that implant-abutment connection design has a significant effect on collagen fiber orientation in peri-implant soft tissues. External hexagon implants mainly display parallel and parallel-oblique fiber arrangements, while Morse taper implants showed a greater proportion of oblique and perpendicular fibers. These results suggest that the geometry and mechanical characteristics of the implant-abutment interface may influence peri-implant connective tissue organization. The absence of significant differences between buccal and lingual aspects within each experimental group suggests that collagen fiber organization was consistent around the implant circumference. Conversely, the significant difference observed between implant connection types indicates that connection design may play a more important role in determining collagen fiber orientation than site-related factors. Therefore, the results should be interpreted as differences between treatment protocols rather than isolated effects of implant-abutment connection geometry. The peri-implant mucosa is composed of a sulcular and junctional epithelium supported by a connective tissue rich in collagen fibers, which exhibits reduced vascularity compared with periodontal tissues (18 - 21 , 27 - 30). The arrangement of these fibers is essential for preserving an effective biological seal and safeguarding peri-implant bone from mechanical and bacterial stresses. In this study, external hexagon implants exhibited collagen fibers mainly oriented parallel to the implant surface, frequently associated with the threaded region and showing little or no insertion into the prosthetic component. This pattern is consistent with previous histological findings (11 , 19 , 20) and may result in reduced resistance to functional loading and bacterial infiltration, given the absence of perpendicular fiber insertion. This finding may be explained by the presence of micromovements and microgaps at the implant-abutment interface in external hexagon systems, which can favor bacterial colonization and inflammatory responses, leading to marginal bone remodeling and apical displacement of the biological width (1 - 6 , 22). Consequently, collagen fibers tend to remain parallel to the implant surface, with limited functional attachment. In contrast, Morse taper implants demonstrated a more favorable pattern of collagen fiber organization, characterized by a higher prevalence of oblique and perpendicular fibers frequently associated with the abutment surface. This finding is likely related to the improved mechanical stability and reduced microgap of conical connections, which minimize micromovements and bacterial infiltration, thereby promoting a more stable peri-implant environment (29 , 30). Additionally, the subcrestal positioning of Morse taper implants may have contributed to increased soft tissue thickness and the formation of a more organized connective tissue collar (28 - 30). The presence of perpendicular collagen fibers and their insertion into the abutment surface suggests a more effective biological seal, enhancing resistance to bacterial penetration and mechanical challenges. Although a previous study from our group evaluated peri-implant soft tissue characteristics around these implant systems, the present investigation specifically focused on quantitative collagen fiber angular distribution under polarized light microscopy. These findings are consistent with previous studies reporting improved soft tissue adaptation and peri-implant stability associated with Morse taper connections (23 - 25). The combination of a thicker connective tissue barrier and a more favorable collagen fiber orientation may provide an additional protective mechanism against peri-implant disease. From a clinical perspective, collagen fiber orientation is a key determinant of peri-implant soft tissue quality (26). The predominance of oblique and perpendicular fibers observed in Morse taper implants may contribute to improved long-term tissue stability and esthetic outcomes, particularly in areas of high esthetic demand. It should be acknowledged that the experimental groups differed not only regarding implant-abutment connection type but also in implant system characteristics and placement depth. Therefore, the observed differences cannot be attributed exclusively to the implant-abutment connection. This study has several limitations that should be considered when interpreting the findings. First, the relatively small sample size may have limited statistical power. Second, the experimental groups differed not only in implant-abutment connection type but also in implant macrodesign and placement depth, introducing potential confounding factors that preclude attributing the observed effects exclusively to connection geometry. Third, the use of an animal model limits the direct extrapolation of the results to clinical conditions in humans. Finally, reproducibility assessment was performed by a single examiner, although a high level of intra-examiner agreement was achieved (ICC = 0.89). Future controlled clinical and histological studies using standardized implant designs and placement protocols are needed to further clarify the specific influence of implant-abutment connection geometry on peri-implant collagen fiber orientation and soft tissue organization.

## Conclusions

Implant-abutment connection design affects collagen fiber orientation in peri-implant soft tissues. Morse taper implants demonstrated a more favorable pattern, with a greater proportion of oblique and perpendicular fibers associated with the abutment surface, while external hexagon implants mainly showed parallel and parallel-oblique fibers related to the implant threads. These results indicate that Morse taper connections may improve the biological seal at the implant-abutment interface.

## Figures and Tables

**Table 1:** Mean values and standard errors (SE) of collagen fiber orientation in the buccal and lingual aspects of Group I and Group II. All values are expressed in degrees.

Variable	Buccal (Mean ± SE)	Lingual (Mean ± SE)
Group I	19.881 ± 3.690	21.918 ± 2.759
Group II	70.197 ± 9.962	54.729 ± 3.527

Abbreviations: SE, standard error.

## Data Availability

The datasets generated and analyzed during the current study are available from the corresponding author upon reasonable request.
